# Supplementary material for: WordCommentsAnalyzer: A windows software tool for qualitative research
Source: F1000Res. 2018 Sep 4;7:536. Originally published 2018 May 3. [Version 2] doi: 10.12688/f1000research.14819.2 (PMC6137411; doi:10.12688/f1000research.14819.2)
Supplement: Supplementary file 3 [file f1000research-7-17614-s0002.tgz › b2a79496-4ada-47c4-9682-a99ce867836d.zip!/Eight Iranian Health Journals/Journal of Research in Rehabilitation Sciences _36.docx]

© Journal of Research in Rehabilitation Sciences . This work is licensed under CC BY-NC 4.0 (https://creativecommons.org/licenses/by-nc/4.0/).

Volume & Issue: دوره 8، شماره 5: 1391

Title: بررسی ارتباط کیفیت زندگی با وضعیت راه رفتن و دامنه حرکتی اندام تحتانی در بزرگسالان مبتلا به فلج مغزی اسپاستیک

http://jrrs.mui.ac.ir/index.php/jrrs/article/view/638

مقدمه: عوارض متعاقب فلج مغزی می‌تواند تأثیرات چشمگیری بر توانمندی‌های فیزیکی و روان‌شناختی بزرگسالان مبتلا به فلج مغزی داشته باشد و کیفیت زندگی آن‌ها را تحت تأثیر قرار دهد. هدف از مطالعه حاضر، تعیین ارتباط بین کیفیت زندگی با وضعیت راه رفتن و دامنه حرکتی در بزرگسالان مبتلا به فلج مغزی بود. مواد و روش‌ها: تحقیق حاضر از نوع تحلیلی- مقطعی بود و بر روی 70 فرد بزرگسال (40 زن و 30 مرد) مبتلا به فلج مغزی اسپاستیک یک طرفه و دو طرفه با میانگین سنی 07/5 ± 24/26 سال، در دامنه سنی 40-20 سال با روش نمونه‌گیری ساده، از افراد در دسترس مراجعه کننده به سه مرکز خدمتگزار به توان‌یابان با نام مجتمع آموزشی نیکوکاری رعد مستقر در شهرهای تهران و کرج وارد مطالعه شدند. کیفیت زندگی، وضعیت راه رفتن و دامنه حرکتی مفاصل زانو و مچ پا به ترتیب با پرسش‌نامه فرم کوتاه کیفیت زندگی سازمان بهداشت جهانی، آزمون طبقه‌بندی عملکردی راه رفتن و گونیامتر دستی ارزیابی شدند. روش‌های آماری مورد استفاده در این مطالعه، آزمون‌های همبستگی Pearson، Spearman، Independent t و Mann-Whitney بودند. یافته‌ها: بین حیطه‌های کیفیت زندگی با وضعیت راه رفتن و دامنه حرکتی دورسی فلکشن مچ پا ارتباطی پیدا نشد، اما حیطه‌های سلامت روانی و سلامت محیط با میزان دامنه حرکتی اکستنشن زانو ارتباط ضعیفی داشتند. همچنین تفاوت معنی‌داری در نمره حیطه‌های کیفیت زندگی بین زنان و مردان وجود نداشت. نتیجه‌گیری: شاید میزان دامنه حرکتی مفصل زانو روی سلامت روان و سلامت محیط بزرگسالان دچار فلج مغزی از نوع اسپاستیک تأثیر داشته باشد. همچنین می‌توان گفت که محدودیت‌های جسمی این افراد به مقدار بسیار کمی کیفیت زندگی آن ها را تحت تأثیر قرار می‌دهد.

Title: بررسی معلولیت شنوایی در بزرگسالان 20 تا 65 ساله کم‌شنوای خفیف تا عمیق با استفاده از پرسش‌نامه فارسی معیار عملکرد ارتباطی دنور (Denver)

http://jrrs.mui.ac.ir/index.php/jrrs/article/view/536

مقدمه: معلولیت شنوایی تأثیرات روحی، روانی و اجتماعی حاصل از کم‌شنوایی است؛ در حالی که آسیب شنوایی، میزان کاهش عملکرد شنوایی بوده و با آزمون‌های شنوایی شناسی اندازه‌گیری می‌شود. پرسش‌نامه‌ها به عنوان ابزارهایی خودارزیاب، یکی از کاربردی‌ترین و در عین حال ساده‌ترین آزمون‌های در دسترس برای شنوایی‌شناسان به منظور بررسی معلولیت‌های ناشی از کم‌شنوایی هستند. مواد و روش‌ها:  گروه هدف این بررسی، بیماران بزرگسال 20 تا 65 سال مبتلا به کم‌شنوایی خفیف تا عمیق بودند. ارزیابی‌های پایه شنوایی شامل اوتوسکوپی، ادیومتری صوت خالص، تیمپانومتری و امتیاز بازشناسی گفتار انجام گرفت و سپس پرسش‌نامه فارسی معیار عملکرد ارتباطی Denver برای همه آن‌ها تکمیل شد و امتیاز آن‌ها از لحاظ ارتباط و معنی‌داری با نتایج آزمون ادیومتری صوت خالص مورد مقایسه قرار گرفت. یافته‌ها: با افزایش میانگین آستانه شنوایی در سه فرکانس 500، 1000 و 2000 هرتز، بیماران امتیاز بالاتری در پرسش‌نامه به دست آوردند (003/0 = P). نتیجه‌گیری: پرسش‌نامه کمی معیار عملکرد ارتباطی Denver از همبستگی مناسبی با آزمون ادیومتری صوت خالص برخوردار بود و می‌توان آن را برای بررسی آسیب و معلولیت شنوایی به کار برد.

Title: زمان آغاز واکداري در همخوان‌هاي انسدادی زبان فارسی

http://jrrs.mui.ac.ir/index.php/jrrs/article/view/565

مقدمه: زمان آغاز واکداري به عنوان يک سرنخ صوتي معتبر جهت افتراق همخوان‌هاي انسدادی واکدار و بي‌واک شناخته شده است و مي‌‌تواند جهت توصيف يا طبقه‌بندي طيفي از اختلالات رشدي، عصبي- حرکتي و يا زباني مورد استفاده قرار گيرد. مقادير هنجار اين متغير در زبان‌هاي متفاوتي استخراج شده و در اين مطالعه نیز مقادير زمان آغاز واکداري در زبان فارسی (گویش معیار) به دست آمد. مواد و روش‌ها: در مطالعه توصیفی- تحلیلی 4 گوینده فارسی زبان که به صورت ساده و در دسترس انتخاب شده بودند، پس از خواندن 42 تک کلمه فارسی که با همخوان‌های انسدادی آغاز می‌شد، در دو مرحله آن‌ها را تکرار کرده و صدایشان ضبط شد. سپس با نرم‌افزار Praat، مقادیر میانگین برای زمان آغاز واکداری با تحلیل طیف صوتی و موج شکل صوتی بر حسب میلی‌ثانیه محاسبه گردید. تفاوت‌های جنسی و نیز تأثیر واکه‌ها بر زمان آغاز واکداری بررسی شد. یافته‌ها: بین مردان و زنان فارسی زبان تفاوت معنی‌داری در زمان آغاز واکداری وجود نداشت (05/0 < P). ارتباط معنی‌داری بین جایگاه تولید همخوان (05/0 > P) با زمان آغاز واکداری وجود داشت. میانگین و انحراف معیار مقادیر زمان آغاز واکداری برای واج‌های /b/، /p/، /t/، /d/، /k/، /g/ و /G/ به ترتیب برابر با (7/12) 41/12، (1/18) 27/78، (9/20) 44/89، (8/9) 63/16، (20) 73/100، (1/9) 34/30 و (69/8) 98/23 و برای دو واج‌گونه [ɟ]، [c] به ترتیب برابر با (7/7) 6/33 و (18) 13/103 میلی‌ثانیه بود. نتیجه‌گیری: در زبان فارسی (گویش معیار)، جنسیت کاربران زبان فارسی تأثیری بر زمان آغاز واکداری همخوان‌های انسدادی آن‌ها نداشت. هر چند واکه‌های پسین و باز و جایگاه تولید، مقدار زمان آغاز واکداری را افزایش می‌داد. زمان آغاز واکداری همخوان‌های انسدادی بی‌واک در دامنه «تأخیر واکداری طولانی» قرار داشته و دمشی محسوب می‌شوند و همخوان‌های انسدادی واکدار در دامنه «تأخیر واکداری کوتاه» قرار دارند.

Title: ارتباط ثبات مرکزی و پارگی لیگامان متقاطع قدامی در فوتبالیست‌ها

http://jrrs.mui.ac.ir/index.php/jrrs/article/view/592

مقدمه: پارگی کامل لیگامان متقاطع قدامی (Anterior cruciate ligament یا ACL) یکی از شایع‌ترین آسیب‌های ورزشکاران است. در مطالعات گذشته، رابطه بین آسیب دیدگی ACL با عواملی مانند جنسیت، سن، نوع فعالیت ورزشی، سطح هورمون‌های جنسی و شلی مفصلی مورد بررسی قرار گرفته است. اگر چه به نظر می‌رسد کاهش تحمل عضلات تنه تحتانی و مرکزی بدن نقش اساسی در آسیب ACL دارد، با این حال این عوامل به اندازه کافی بررسی نشده‌اند. هدف اصلی مطالعه حاضر، بررسی ارتباط ثبات مرکزی و پارگی لیگامان متقاطع قدامی در فوتبالیست‌ها بود. مواد و روش‌ها: در این مطالعه مقطعی، تحمل عضلات اکستنسور، فلکسور و لترال فلکسور تنه در 30 فوتبالیست حرفه‌ای شامل 15 نفر مبتلا به پارگی ACL (میانگین سن = 4/23 سال) و 15 نفر سالم (میانگین سن = 7/21 سال) اندازه‌گیری شد. برای اندازه‌گیری تحمل این عضلات در هر دو گروه و به وسیله کرونومتر دیجیتال، از تست‌های McGill و Biering-Sorensen استفاده گردید. یافته‌ها: تحمل عضلات اکستنسور، فلکسور و لترال فلکسور تنه در فوتبالیست‌های مبتلا به پارگی ACL در مقایسه با فوتبالیست‌های سالم به صورت معنی‌داری کمتر بود (05/0 > P). نتیجه‌گیری: کاهش تحمل عضلات تنه شاید عاملی جهت افزایش خطر پارگی ACL در فوتبالیست‌ها است. این موضوع باید در مطالعات مقایسه‌ای آینده تأیید گردد. افزایش تحمل این عضلات به احتمال زیاد سبب کاهش خطر آسیب ACL می‌شود.

Title: تأثير فعاليت‌هاي حركتي ظريف بر رشد مهارت‌هاي ‌ديداري- ادراكي افراد با نشانگان داون

http://jrrs.mui.ac.ir/index.php/jrrs/article/view/619

مقدمه: رشد مهارت‌های دیداری- ادراکی در کودکان با نشانگان داون اغلب تأخیر قابل ملاحظه‌ای دارد. هدف این مطالعه، بررسی تأثير فعاليت‌هاي حركتي ظريف بر رشد مهارت‌هاي ‌ديداري- ادراكي افراد با نشانگان داون بود. مواد و روش‌ها: مطالعه تجربی حاضر بر روی 18 نفر از افراد 13-9 ساله با نشانگان داون که به روش نمونه‌گیری در دسترس انتخاب شده بودند، انجام شد. شرکت کنندگان با استفاده از جدول اعداد تصادفی به دو گروه 9 نفره تقسیم شدند . افراد گروه مداخله طی 24 جلسه به انجام فعالیت‌های حرکتی ظریف منظم پرداختند. شرکت کنندگان قبل و بعد از مرحله مداخله به وسیله نسخه تجدید نظر شده آزمون مهارت‌های دیداری- ادراکی (TVPS-R) آزموده شدند. تحلیل آماری داده‌ها با استفاده از آزمون‌های Kolmogorov-Smirnov، Independent t، Paired t و 2c انجام شد. یافته‌ها: تفاوت معنی‌داری میان نمرات گروه مداخله، قبل و بعد از انجام فعالیت‌های حرکتی ظریف وجود داشت (05/0 >P )، ولی این تفاوت در گروه مقایسه معنی‌دار نبود. همچنین تفاوت میان نمرات دو گروه، بعد از انجام مداخله معنی‌دار بود (014/0 = P). نتیجه‌گیری: انجام فعالیت‌های حرکتی ظریف باعث رشد مهارت‌های دیداری- ادراکی افراد مبتلا به نشانگان داون می‌شود و این می‌تواند به معنای لزوم برنامه‌ریزی مداخلات حرکتی ظریف برای کودکان با نشانگان داون و آموزش والدین آنان در این زمینه باشد.

Title: مقايسه روش‌های متفاوت ارایه برنامه‌هاي تلويزيوني بر زبان دريافتی ناشنوايان 15 تا 25 ساله شهر اصفهان

http://jrrs.mui.ac.ir/index.php/jrrs/article/view/391

چکيده مقدمه: نقص شنوایی از جمله اختلالاتی است که بر مهارت‌های زبانی و ارتباطی مبتلایان تأثیر شدیدی دارد و تاکنون در زمینه نحوه ارایه برنامه‌های تلویزیونی بر زبان دريافتی ناشنوايان کمتر پژوهشی صورت گرفته است. این پژوهش با هدف، مقايسه ارایه سه روش متفاوت برنامه تلويزيوني بر ميزان زبان دریافتی (ادراکی) ناشنوايان در دو مرحله دریافتی و توجهي در شهر اصفهان انجام گرفت. مواد و روش‌ها: پژوهش حاضر از نوع آزمايشي بود و از طرح پس‌آزمون با گروه شاهد استفاده شد. بدين منظور تعداد 48 نفر از ناشنوايان با روش تصادفي انتخاب و به تصادف در سه گروه قرار داده شدند. براي جمع‌آوري اطلاعات از آزمون محقق ساخته كه روايي و پايايي آن مورد تأييد قرار گرفته بود، استفاده شد. داده‌ها با استفاده از روش‌هاي آماري ANOVA و ANCOVA و با بهره‌گيري از نرم‌افزار SPSS نسخه 18 تجزيه و تحليل گرديد. يافته‌ها: بين عملکرد زبان دریافتی هر سه گروه از ناشنوايان در دريافت برنامه‌هاي تلويزيوني و در هر دو مرحله دریافتی و توجهی تفاوت معني‌دار آماری وجود دارد (05/0 > P). نتيجه‌گيري: بنابراین مي‌توان گفت ارایه برنامه‌هاي تلويزيوني با روش تركيبي، بيشترين تأثير را بر ميزان زبان دریافتی (ادراکی) ناشنوايان دارد و به مسؤولان رسانه‌های دیداری به خصوص صدا و سیما پیشنهاد می‌شود که در ارایه برنامه‌های تلویزیونی از روش ترکیبی استفاده نمایند

Title: بررسی ویژگی‌های طیفی در افراد دارای شکاف کام و مقایسه آن با همتایان طبیعی

http://jrrs.mui.ac.ir/index.php/jrrs/article/view/593

مقدمه: پرخیشومی یک اختلال در تشدید است که در آن صدا در حین گفتار وارد حفره بینی می‌شود و به خصوص در واکه‌ها بیشتر قابل درک می‌باشد. اگر چه ارزيابي ادراكي، استانداردي طلايي براي ارزيابي پرخیشومی است، ولی دارای مشکلاتی است که از اعتبار آن می‌کاهد. تلاش‌های زیادی در جهت رشد و گسترش ارزیابی‌های دستگاهی و آکوستیکی دارای روایی و اعتبار انجام شده که می‌تواند مکمل ارزیابی‌های ادراکی باشد. هدف از مطالعه حاضر، به کارگیری و گسترش تجزیه و تحلیل آکوستیک طیف 3/1 اکتاو جهت ارزیابی کمی پرخیشومی در افراد شکاف کام دارای پرخیشومی و مقایسه آن با افراد طبیعی بود.  مواد و روش‌ها: مطالعه توصيفی- تحليلي- مقطعي حاضر بر روی 20 فرد طبیعی (6 دختر و 14 پسر) با میانگین سنی 7/2 ± 01/8 سال و 10 فرد دارای شکاف کام (3 دختر و 7 پسر) با میانگین سنی 2/2 ± 17/8 سال انجام شد. افراد دارای شکاف کام  توسط دو آسیب‌شناس گفتار و زبان ارزیابی شدند که شدت پرخیشومی این افراد متوسط تا شدید بود. از این افراد خواسته شد که واکه /i/ را به صورت کشیده تولید نمایند. نمونه‌های به دست آمده از نظر ارزیابی آکوستیکی و با نرم‌افزار Praat نسخه 5.2.2.7 مورد بررسی قرار گرفت. سپس این داده‌ها با استفاده از نرم‌افزار SPSS نسخه 16 و آزمون های آماریt  Independent مورد تجزیه و تحلیل قرار گرفت. یافته‌ها: دامنه پرخیشومی در افراد فارسی زبان نسبت به افراد طبیعی به طور معنی‌داری در فرکانس‌های مرکزی 630، 800، 1000 و 1600 بیشتر بود. نتیجه‌گیری: ارزیابی طیف 3/1 اکتاو در فرکانس‌های مرکزی یاد شده شاخص خوبی برای تبیین حضور سازه خیشومی در گفتار افراد دارای شکاف کام است. همچنین استفاده از تجزیه و تحلیل آکوستیک طیف 3/1 اکتاو جهت بررسی ویژگی‌های تشدید به صورت کمی مفید می‌باشد.

Title: تأثیر تمرینات جسمانی بر میزان فرسودگی شغلی پرستاران

http://jrrs.mui.ac.ir/index.php/jrrs/article/view/548

مقدمه: فرسودگی شغلی، سندرمی مشتمل بر خستگی عاطفی، مسخ شخصیت و عدم توانایی فردی می‌باشد. پرستاران به دلیل مواجهه با استرس‌های فیزیکی، روانی و هیجانی به شدت در معرض فرسودگی شغلی می‌باشند. هدف از تحقیق حاضر، بررسی تأثیر 8 هفته تمرینات جسمانی بر میزان فرسودگی شغلی پرستاران بود. مواد و روش‌ها: بدین منظور از پرسش‌نامه Maslash and Jackson استفاده گردید. این پرسش‌نامه در بین 24 نفر از پرستاران در دو مرحله، یک بار قبل از شروع و دیگر بار بعد از اتمام 8 هفته تمرینات ورزشی توزیع گردید و سپس داده‌ها توسط نرم‌افزار SPSS مورد تجزیه و تحلیل قرار گرفت. یافته‌ها: میانگین مربوط به خستگی عاطفی و مسخ شخصیت بعد از 8 هفته تمرینات اصلاحی کاهش یافت (01/0 > P). همچنین میانگین مربوط به موفقیت فردی افزایش یافت (001/0 > P). در مورد اولویت‌بندی تغییر عوامل نتایج آزمون Friedman نشان داد، عامل خستگی عاطفی تغییرات بیشتری نسبت به توانایی فردی و مسخ شخصیت دارد (05/0 > P). نتیجه‌گیری: میزان فرسودگی شغلی در پرستاران در سطح متوسط و رو به بالایی است و با توجه به تأثیر آن در عملکرد شغلی، خانوادگی، فردی و نیز اثر آن بر غیبت از کار و بازنشستگی زود هنگام، باید بر روش‌های پیشگیری یا کاهش آن توسط تمرینات ورزشی تأکید داشت.

Title: بررسی تأثیر روش دوسا (Dohsa-hou) در کاهش علایم بیش‌فعالی نقص توجه و افزایش مهارت‌های اجتماعی دانش‌آموزان مبتلا به بیش‌فعالی و نقص توجه 6 تا 11 سال

http://jrrs.mui.ac.ir/index.php/jrrs/article/view/525

مقدمه: پژوهش حاضر به منظور بررسی تأثير توان‌بخشي رواني دوساهو بر کاهش علایم بیش‌فعالی نقص توجه و افزایش مهارت‌های اجتماعی انجام شد. مواد و روش‌ها: مطالعه حاضر به شیوه شبه تجربي با طرح پیش‌آزمون- پس‌آزمون با گروه شاهد و مورد اجرا گردید. 24 کودک مبتلا به نقص توجه همراه با بیش‌فعالی از مرکز کاردرمانی برنا به صورت نمونه در دسترس انتخاب و به صورت تصادفی به دو گروه شاهد و مورد  تقسیم شدند. قبل از شروع دوره، والدین کودکان موظف به تکمیل پرسش‌نامه‌های مشکلات رفتاری کودکان Conners (Conners parent rating scale CPRS)، علایم مرضی کودکان (Child symptom inventory-4 یا CSI-4) و پرسش‌نامه مهارتهای اجتماعی (فرم والدین) شدند و در پایان دوره دوباره این ارزیابی‌ها تکرار گردید. مدت اجرای دوساهو در گروه آزمایش 12 جلسه به صورت هفتگی و هر هفته دو جلسه در نظر گرفته شد. سپس داده‌های حاصل از پرسش‌نامه‌ها با نرم‌افزار SPSS نسخه 16 (Version 16, SPSS Inc., Chicago, IL., USA) به روش ANCOVA تجزیه و تحلیل گردید و همه فرضیه‌ها مورد تأیید قرار گرفت. یافته‌ها: یافته‌های حاصل از ANCOVA حاکی از تأثیر معنی‌دار توان‌بخشی روانی دوساهو بر تعاملات اجتماعی و علایم بیش‌فعالی در کودکان مبتلا به اختلال بیش‌فعالی نقص توجه بود این پژوهش نشان داد، اجرای تکنیک‌های دوساهو مهارت‌های اجتماعی و توجه کودکان مبتلا به اختلال بیش‌فعالی نقص توجه را افزایش و بیش‌فعالی و تکانشگری را در این کودکان کاهش می‌دهد (001/0 = P). نتیجه‌گیری: با استفاده از روش توان‌بخشی روانی و به کارگیری تلاش و اراده در کودکان مبتلا به اختلال بیش‌فعالی نقص توجه می‌توان به آن‌ها در افزایش مهارت‌های اجتماعی و کاهش علایم بیش‌فعالی یاری رساند، بنابراین می‌توان به خانواده‌ها و دیگر مسؤولین تعلیم و تربیت استثنایی، استفاده از این روش را جهت بهبود تعاملات اجتماعی و علایم بیش‌فعالی این کودکان پیشنهاد کرد.

Title: بررسی تأثیر تخریب ملانوسیت‌های گوشی بر عملکرد شنوایی و دهلیزی در بیماران مبتلا به ویتیلیگو

http://jrrs.mui.ac.ir/index.php/jrrs/article/view/813

مقدمه: ویتیلیگو بیماری است که منجر به تخریب سلول‌های ملانوسیت در سطح بدن می‌گردد و بارزترین یافته بالینی آن ایجاد لکه‌های سفید رنگ پوستی می‌باشد، اما به دلیل حضور گسترده‌ ملانوسیت‌ها در سیستم شنوایی و دهلیزی و نقش فیزیولوژیکی خاص آن، احتمال درگیری این سیستم‌ها نیز در طی فرایند بیماری وجود دارد. مواد و روش‌ها: مطالعه مقطعی حاضر بر روی 21 بیمار مبتلا به ویتیلیگو (12 مؤنث و 9 مذکر)، با میانگین سنی 06/8 ± 14/30 سال (44-19 سال) و 21 نمونه هنجار (11 مؤنث و 10 مذکر)، با میانگین سنی93/7 ± 19/31 سال (44-18 سال) انجام گرفت. آزمون‌های ادیومتری تون خالص (Pure tone audiometry یا PTA)، پاسخ شنیداری ساقه مغز (Auditory brainstem response یا ABR) و پتانسیل عضلانی برانگیخته دهلیزی (Vestibular evoked myogenic potentials یا VEMP) در تمام این افراد مورد ارزیابی قرار گرفت. یافته‌ها: افت شنوایی حسی- عصبی فرکانس بالا در 8 بیمار (09/38 درصد) مشاهده گردید. تحلیل نتایج آزمایش ABR نشان دهنده افزایش معنی‌دار نهفتگی موج III در 10 بیمار (61/47 درصد) بود که در گوش راست و چپ تفاوت آماری معنی‌داری را کسب نمود (به ترتیب 024/0 = P و 001/0 = P) و نیز افزایش در نهفتگی بین قله‌ای I-III در 6 بیمار (57/28 درصد) ثبت گردید. در آزمایش VEMP در گوش چپ 1 بیمار (76/4 درصد) موجی ثبت نگردید و افزایش در نهفتگی موج 13p در 5 بیمار (80/23 درصد) ثبت گردید که سطح معنی‌دار آماری را کسب نمود (001/0 = P). نتیجه‌گیری: با توجه به نقش آنتی‌اکسیدانی، بازیافت یونی و نیمه هدایتی ملانوسیت‌ها در سیستم شنوایی و دهلیزی، احتمال درگیری این سیستم‌ها در طی فرایند بیماری ویتیلیگو که کل ملانوسیت‌های سطح بدن مورد تهاجم قرار می‌گیرند، وجود دارد. در کل، در این افراد نتایج غیر طبیعی آزمون‌های PTA و ABR بیشتر از آزمون VEMP بود که می‌تواند از تمرکز کمتر ملانوسیت‌ها در ارگان دهلیزی ساکول نسبت به ارگان‌های شنوایی ناشی شده باشد.

Title: مقایسه قدرت و استقامت عضلات ثبات دهنده تنه در افراد مبتلا به سندرم درد مفصل پاتلوفمورال و افراد سالم

http://jrrs.mui.ac.ir/index.php/jrrs/article/view/703

مقدمه: تنه پایه‌ای برای حرکات چرخشی در كمربند شانه‌اي و لگني است و ثبات ستون مهره‌ها، لگن و اندام تحتانی را حین حرکت حفظ می‌کند. ثبات دینامیک تنه توسط عضلات این ناحیه تأمین می‌شود و اختلال در عملکرد عضلات این ناحیه می‌تواند باعث بروز مشکلات عضلانی- اسکلتی در سایر قسمت‌های بدن گردد. یکی از مشکلات شایع زانو، سندرم درد مفصل پاتلوفمورال (Patello femoral pain syndrome یا PFPS) است. با توجه به شیوع این بیماری در افراد جوان و نقش عضلات تنه به عنوان عامل ثبات دهنده قسمت‌هاي پروگزیمال برای حرکت بخش‌هاي دیستال، ضروری به نظر می‌رسد که تأثیر عضلات ثبات دهنده تنه برای سندرم PFPS بررسی شود. مواد و روش‌ها: 15 بیمار مبتلا به PFPS و 15 فرد سالم مورد ارزیابی قرار گرفتند. قدرت و استقامت عضلات تنه شامل اکستانسورها، فلکسورها و فلکسورهای جانبی راست و چپ تنه بین دو گروه مورد مقایسه قرار گرفت. همچنین در گروه بیمار شدت درد از طریق معیار آنالوگ چشمی (Visual analogue scale یا VAS) اندازه گرفته شد. یافته‌ها: استقامت عضلات ثبات دهنده تنه در گروه مبتلا به سندروم درد پتلوفمورال به طور معنی‌داری در عضلات فلکسور (001/0 =P )، اکستانسور (005/0 =P )، فلکسورهای جانبی راست (016/0 =P ) و چپ (058/0 =P ) كمتر از گروه سالم بود. بین شدت درد و کاهش استقامت عضلات تنه همبستگي معنی‌داری مشخص نگردید. نتیجه‌گیری: در درمان بیماران مبتلا به PFPS علاوه بر ورزش‌های معمول، توجه به قدرت و استقامت عضلات تنه ضروری به نظر مي‌رسد.

Title: بررسی فراوانی ناروانی در کلمات و ناکلمات در بزرگسالان فارسی زبان مبتلا به لکنت

http://jrrs.mui.ac.ir/index.php/jrrs/article/view/529

مقدمه: درک بهتر ماهیت پیچیده و چند وجهی لکنت مستلزم بررسی جنبه‌های مختلف آن می‌باشد، بنابراین مطالعه لکنت از جنبه‌های زبانی و بررسی تأثیر عوامل زبان‌شناختی در آن از اهمیت ویژه‌ای برخوردار است. هدف این پژوهش، بررسی فراوانی بروز ناروانی در کلمات و ناکلمات در بزرگسالان مبتلا به لکنت فارسی زبان بود. مواد و روش‌ها: در مطالعه حاضر، 15 فرد بزرگسال مبتلا به لکنت (15 سال به بالا) فارسی زبان مورد بررسی قرار گرفتند. برای بررسی ابتدا فهرست کلمات و ناکلمات تهیه گردید. به این ترتیب که 60 کلمه دو تا پنج هجایی با فراوانی وقوع متنوع در زبان فارسی انتخاب شد و سپس 60 ناکلمه بر اساس کلمات و تغییر حداقل 50 درصد واج‌های کلمه ساخته شد. افراد شرکت کننده کلمات و ناکلمات را تکرار کردند و داده‌ها ضبط و جمع‌آوری گردید. سپس تجزیه و تحلیل داده‌ها  توسط نرم‌افزار SPSS انجام شد. یافته‌ها: فراوانی ناروانی در کلمات و ناکلمات به طور معنی‌داری متفاوت است و ناکلمات در مقایسه با کلمات در بزرگسالان مبتلا به لکنت افزایش معنی‌داری دارد (05/0 > P). نتیجه‌گیری: نتایج حاصل از این پژوهش نشان دهنده افزایش معنی‌دار میزان ناروانی گفتار در تکرار ناکلمات در مقایسه با تکرار کلمات در بزرگسالان مبتلا به لکنت فارسی زبان می‌باشد. همچنین نوع ناروانی‌ها در هر دو فهرست کلمات و ناکلمات بیشتر از نوع گیر (ناروانی‌های لکنتی) می‌باشد.

Title: تأثیر نمایش درمانی مبتنی بر مهارت اجتماعی رفتار سازگارانه دختران کم‌توان ذهنی با هوش‌بهر 70-55 در دامنه سنی 30-14 سال

http://jrrs.mui.ac.ir/index.php/jrrs/article/view/558

مقدمه: حدود 2 درصد از کودکان سنین مدرسه را افراد عقب‌مانده ذهنی خفیف تشکیل می‌دهند. این افراد می‌توانند از توانایی‌های خود جهت تأمین نیازهای خود و انتظارات جامعه به کفایت بهره گیرند. بنابراین برنامه‌های آموزشی باید به نحوی باشد تا بتوانند روابط مفید و مؤثر اجتماعی برقرار کنند. پژوهش حاضر با هدف، بررسی تأثیر نمایش درمانی بر رفتار سازگارانه دختران کم‌توان ذهنی با هوش‌بهر 70-55 در دامنه سنی 30-14 سال انجام شد. مواد و روش‌ها: مطالعه توصیفی- تحلیلی حاضر بر روی کلیه معلولین دختر (14 نفر) مرکز توان‌بخشی کوثر شهرستان بیرجند، با هوش‌بهر 70-55 و در دامنه سنی 30-14 سال صورت گرفت. ابتدا ارزیابی رفتار سازگارانه توسط مقیاس Vaynlnd انجام گرفت، سپس با روش نمایش درمانی به مدت 12 جلسه، هفته‌ای 2 بار و هر جلسه 5/1 ساعت ، تحت آموزش مهارت‌های اجتماعی قرار گرفتند. نمره رفتارهای سازگارانه آنان بعد از برنامه‌های نمایش درمانی نیز ارزیابی گردید. میانگین نمرات از طریق آزمون آماریPaired t  مورد مقایسه قرار گرفت. یافته‌ها: تحلیل داده‌ها نشان داد که نمرات رفتارهای سازگارانه قبل و بعد از مداخله اختلاف معنی‌داری داشته و تغییرات مثبت بوده‌اند(002/0 = P). نتیجه‌گیری: نمایش درمانی در نمونه مورد مطالعه موجب بهبود رفتار سازگارانه آنان گردید.

Title: ارزیابی ارگونومیک خطر ابتلا به اختلالات عضلانی- اسکلتی معلمان به روش QEC و تأثیر 8 هفته حرکات اصلاحی منتخب و مداخلات ارگونومی شغلی بر میزان مواجهه آن‌ها

http://jrrs.mui.ac.ir/index.php/jrrs/article/view/532

مقدمه: ناراحتی‌های عضلانی- اسکلتی بخش عمده‌ای از بیماری‌های شغلی را به خود اختصاص می‌دهند. هدف از تحقیق حاضر، ارزیابی ارگونومیکی خطر ابتلا به اختلالات عضلانی- اسکلتی معلمان به روش ارزیابی سریع مواجهه (Quick exposure check یا QEC) و بررسی تأثیر 8 هفته تمرینات اصلاحی منتخب و مداخلات ارگونومی شغلی بر میزان مواجهه آن‌ها بود. مواد و روش‌ها: در این مطالعه از پرسش‌نامه Nordic و روش ارزیابی سریع مواجهه (QEC) در 4 وظیفه کاری معلمان استفاده شد. پرسش‌نامه Nordic بین 230 نفر از معلمان توزیع گردید و از این تعداد 30 نفر که دارای اختلالات عضلانی- اسکلتی بودند شناسایی و به عنوان نمونه‌های تحقیق انتخاب شدند. یافته‌ها: 7/72 درصد از معلمان، کار در محیط شغلی را عامل اصلی ایجاد اختلالات عضلانی- اسکلتی گزارش کردند و 75 درصد از وظایف مورد مطالعه معلمان در سطح بالای خطر قرار داشتند. تفاوت معنی‌داری در وظایف معلمان مشاهده شد (05/0 > P)؛ به طوری که از 4 وظیفه مورد بررسی در معلمان، وظیفه تدریس و نوشتن روی تابلوی کلاس (6/57 درصد) و نشستن پشت میز کلاس (7/56 درصد) بیشترین امتیاز خطر را به خود اختصاص داد. نظارت و ديدن تکاليف بر روي میز دانش‌آموزان (8/54 درصد) و تدريس و نظارت بر کلاس به حالت ايستاده (1/43 درصد) در مراتب بعدی خطر قرار گرفتند. به دنبال 8 هفته تمرینات اصلاحی و مداخلات ارگونومی، کاهش معنی‌داری در میزان خطر ابتلا به اختلالات عضلانی- اسکلتی در 4 وظیفه کاری معلمان مشاهده شد (05/0 > P). نتیجه‌گیری: بالا بودن سطح خطر، نشانه آسیب‌زا بودن شرایط و محیط کار در مدارس بود که اصلاح تکنیک در وظایف گفته شده و همچنین حرکات اصلاحی منتخب موجب کاهش سطح خطر شد.

Title: بررسي ميانگين طول گفته (MLU) در كودكان 2/5 تا 5/5 ساله فارسي زبان اصفهان

http://jrrs.mui.ac.ir/index.php/jrrs/article/view/654

مقدمه: یکی از شاخص‌های کمی سنجش توانمندی نحو در كودكان پيش‌دبستاني، ميانگين طول گفته (Mean length of utterance یا MLU) است كه در ايجاد يك تقسيم‌بندي مرحله‌اي از روند رشد نحوي زبان كمك مي‌كند. نظر به این که هنوز اطلاعات کاملی از این شاخص درباره کودکان فارسی زبان در دست نیست، در اين بررسي MLU در كودكان پیش‌دبستانی فارسی زبان و حساسیت آن به سن بررسي شد. مواد و روش‌ها: در يك مطالعه مقطعی بر روي نمونه‌هاي زباني 171 كودك طبيعي 2/5 تا 5/5 ساله شهر اصفهان که به شيوه تركيبي، انتخاب و در بازه‌های سنی شش ماهه دسته‌بندي شدند، پس از یک مصاحبه و بازی غیر رسمی با کودک برای تعیین وضعیت رشد طبیعی شناخت و زبان هر کودک، نمونه‌هاي زباني تعامل آن‌ها با آزمونگر، ضبط و سپس 75 گفته واضح، متوالي و بیش از تک کلمه، برای محاسبه MLU در نظر گرفته شد. ميانگين و انحراف معيار MLU كودكان بر حسب تکواژ در فواصل سني 6 ماهه و حساسیت آن به رشد سنی نیز بررسی گرديد. یافته‌ها: ميانگين  MLUبا افزایش سن، افزایش يافت كه بخشي از اين تغيير از لحاظ آماري معنی‌دار بود. مقايسه تفاوت بين دو بازه سني 3 تا 3/5 ساله و 3/5 تا 4 ساله با دو بازه سني بعدي نشان دهنده پیشرفت بیشتر رشد نحوی گروه اول نسبت به دو گروه دوم است و رابطه بين سن به ماه و ميانگين MLU بر حسب تكواژ نیز معني‌دار بود. نتیجه‌گیری: میانگین MLU در پژوهش فعلی، بیشتر از تحقیقات گزارش شده انگلیسی و میزان رابطه آن با سن، کمتر به دست آمده است. عمده این تفاوت در نتایج، به تفاوت‌های ساخت‌شناسی بین دو زبان مربوط می‌شود، اما نتایج حاصل از بررسی حاضر، به نتایج دو تحقیق فارسی زبان بسیار نزدیک است. حساس بودن مقیاس MLU به سن بر اساس نتایج این بررسی، نشان دهنده قابلیت نسبی آن به عنوان یک شاخص رشد زبانی در زبان فارسی است.

Title: ارتباط میان شاخص تقارن با پارامترهای نوسان وضعیتی در تکلیف پویا و آزمون های تعادل عملکردی در بیماران همی پارزی مزمن راست و چپ (مطالعه مقدماتی)

http://jrrs.mui.ac.ir/index.php/jrrs/article/view/740

مقدمه: استقلال در تکلیف پویای خم شدن و برداشتن شی از روی زمین اهمیت زیادی در فعالیت‌های روزمره زندگی دارد. عدم تقارن در تحمل وزن از عوامل مؤثر در بر هم خوردن تعادل و پایداری وضعیتی در انجام این تکلیف در بیماران همی‌پارزی (Hemiparesis) به شمار می‌رود. این مطالعه با هدف، بررسی ارتباط شاخص تقارن با نوسانات وضعیتی در این تکلیف پویا و آزمون‌ های تعادل عملکردی در بیماران همی‌پارزی مزمن راست و چپ انجام شد. مواد و روش‌ها: 6 همی‌پارزی راست و 11 همی‌پارزی چپ با نمونه‌گیری غیر احتمالی ساده در این مطالعه شرکت کردند. تقارن در تحمل وزن توسط ترازو، پارامترهای نوسان وضعیتی در تکلیف پویا توسط دستگاه صفحه نیرو و تعادل عملکردی توسط آزمون‌های Functional Reach و Timed up and go سنجیده شد و به منظور بررسی ارتباط میان متغیرها از ضریب همبستگی Pearson استفاده گردید. یافته‌ها: میانگین (انحراف معیار) زمان گذشته از ضایعه بیماران همی‌پارزی راست و چپ به ترتیب (06/43) 33/49 و (13/91) 36/75 ماه بود. شاخص تقارن همبستگی بالا تا بسیار بالایی (925/0-865/0 = r) با متوسط سرعت نوسانات مرکز فشار در هدف راست- دور و با کل مسیر طی شده نوسانات در تمام هدف‌های نزدیک و هدف راست- دور در بیماران همی‌پارزی راست و همبستگی بالایی (716/0- = r) با حداکثر جابجایی قدامی- خلفی نوسانات در هدف چپ- نزدیک در بیماران همی‌پارزی چپ نشان داد؛ در حالی که هیچ ارتباط معنی‌داری با حداکثر جابجایی قدامی- خلفی نوسانات در بیماران همی‌پارزی راست و با حداکثر جابجایی داخلی- خارجی نوسانات در بیماران همی‌پارزی چپ نداشت. همچنین شاخص تقارن با آزمون Functional Reach در بیماران همی‌پارزی چپ همبستگی متوسطی (623/0- = r) داشت، اما در بیماران همی‌پارزی راست هیچ ارتباطی با آزمون‌های تعادلی نداشت. نتیجه‌گیری: با توجه به تفاوت ارتباط شاخص تقارن با پارامترهای نوسان وضعیتی در تکلیف پویا و آزمون‌های تعادل عملکردی در بیماران همی‌پارزی راست و چپ، می توان‌گفت که این دو گروه از راهکارهای متفاوتی در انجام این تکلیف استفاده می‌کنند.

Title: كودكان دچار معلوليت و موانع حضور منظم در جلسات توانبخشی

http://jrrs.mui.ac.ir/index.php/jrrs/article/view/474

مقدمه: والدين كودكان معلول با بردن منظم فرزند خود به جلسات توان‌بخشی نقش بسزايي در موفقيت برنامه درمان فرزندشان ايفا می‌كنند، اما همه والدين به طور منظم فرزند خود را طبق وقت قبلی برای شرکت در جلسات توان‌بخشی به مراکز نمی‌برند. هدف از انجام اين مطالعه، تعيين موانع پیش روی والدين در بردن منظم کودکان خود به جلسات توان‌بخشی بود. مواد و روش‌ها: در مطالعه مقطعي حاضر با والدين 92 نفر از كودكان دچار معلوليت که در مراکز سرپایی كاردرماني، گفتاردرماني و فيزيوتراپي شهر بيرجند خدمات توان‌بخشی دریافت می‌کردند، مصاحبه شد. اين مصاحبه بر اساس پرسش‌نامه محقق ساخته متشکل از سؤالات باز و بسته صورت گرفت. روايي پرسش‌نامه با کسب نظر 11 نفر از صاحب‌نظران و محاسبه نسبت روایی محتوایی به روش Lawshe، 73/0 تأیید شده بود و پايايي آن بر اساس نتايج آزمون- باز آزمون، 89/0 بود. داده‌هاي جمع‌آوري شده به كمك نرم‌افزار SPSS نسخه 16 مورد تجزيه و تحليل قرار گرفت. بر حسب مورد از آزمون‌های 2c، Independent t و يا Fisher exact test استفاده شد. مقدار 05/0 > P به عنوان سطح معنی‌داري تفاوت‌ها در نظر گرفته شد. يافته‌ها: 44 نفر (8/47 درصد) از والدین به طور مرتب فرزند خود را برای شرکت در جلسات توان‌بخشی به مراکز می‌آوردند. بر اساس پاسخ والدين به سؤالات بسته پرسش‌نامه، از ميان 28 مانع بالقوه مورد سؤال، "هزينه زياد خدمات توان‌بخشي"، "فاصله زياد خانه تا مركز توان‌بخشي"، "دشواري حمل كودك به مركز توان‌بخشي"، "شرايط بد آب و هوايي در فصولي از سال" و "فرصت كافي نداشتن" به ترتیب بيشترين ممانعت را براي حضور منظم در جلسات درمان ايجاد كرده بودند. در بررسی ارتباط بين مواجهه با هر يک از 28 مانع مورد سؤال با حضور منظم در جلسات توان‌بخشی، تنها بين محدوديت ساعات كار مراكز توان‌بخشي و حضور منظم در جلسات توان‌بخشی ارتباط آماري معنی‌داري به دست آمد (879/0-037/0 = Cl، 181/0 = OR، 02/0 = P). نتيجه‌گيري:  با توجه به اثرات مثبت حضور منظم در جلسات توان‌بخشی بر پیشرفت کودکان معلول و با در نظر گرفتن نتایج حاصل از این پژوهش، انجام پژوهش‌های مداخله‌ای توصیه می‌گردد که طی آن اثر رفع هر يک از موانع فوق بر پيروی والدين از توصیه درمانگران جهت حضور منظم در جلسات توان‌بخشی مورد بررسی قرار گيرد.

Title: مقایسه سرعت حرکتی در کودکان و نوجوانان نابینا و همتایان سالم

http://jrrs.mui.ac.ir/index.php/jrrs/article/view/306

مقدمه: آسیب دیداری روی تمام جنبه‌های رشدی کودک تأثیر می‌گذارد. هدف از این مطالعه، مقایسه سرعت حركتي بین کودکان و نوجوانان نابینا و همتایان سالم بود. مواد و روش‌ها: پژوهش مقطعی- تحلیلی حاضر با روش نمونه‌گیری در دسترس بر روی 30 دانش‌آموز نابینا و 30 دانش‌آموز نيز به عنوان همتايان سالم از مدارس شهر تهران انجام شد. تكليف ضربه زدن با انگشت به عنوان آزمون عصب روان‌شناختي، جهت ارزیابی مورد استفاده قرار گرفت. داده‌های جمع‌آوری شده به کمک آزمون Independent t تجزیه و تحلیل شد. یافته‌ها: بین دو گروه از نظر سرعت حركتي دست راست(001/0 ≥ P)، دست چپ (0001/0 ≥ P) و هر دو دست (0001/0 ≥ P) تفاوت معنی‌داری وجود دارد. نتیجه‌گیری: وقتی سیستم دیداری آسیب دیده است، مشکلاتی در توانایی حرکتی نیز ظاهر می‌شود. در ارزیابی بالینی و مداخلات مربوط به گروه‌های مبتلا به نابینایی، باید به همزمانی آسیب‌های دیداری و نقص‌های حرکتی توجه شود.

Title: نقایص حافظه فعال در کودکان دارای لکنت

http://jrrs.mui.ac.ir/index.php/jrrs/article/view/521

مقدمه: لکنت با توقف‌های زیاد و طولانی مدت در جریان روان گفتار همراه است. این توقف‌ها به صورت تکرار صداها، هجاها، کلمات تک هجایی، کشیده‌گویی صداها و قفل و گیر در مسیر جریان هوای گفتاری یا جریان صداسازی گفتار دیده می‌شود. تاکنون نظریه‌های مختلفی در خصوص چگونگی ایجاد و عوامل مؤثر بر لکنت مطرح شده‌اند، همچنین نقایص زبانی مختلفی در زمینه لکنت مورد بررسی قرار گرفته‌اند. یکی از حیطه‌های زبانی که به تازگی مورد توجه قرار گرفته است، حافظه فعال می‌باشد. حافظه فعال در کودکان مبتلا به لکنت در تحقیقات مختلفی مورد بررسی قرار گرفته است. هدف از مطالعه حاضر، مروری بر یافته‌های تحقیقات انجام شده در این زمینه می‌باشد. مواد و روش‌‌ها: با استفاده از کلید واژه‌‌های "Stuttering"، "Working memory" و "Nonword repetition" مقالات منتشر شده در این مورد، در بانک‌های اطلاعاتی ISI، Science Direct، PubMed، Scholar Google، Proquest، Scopus و بانک‌های اطلاعاتی داخل کشور در بازه زمانی 1990 تا 2012، مورد بررسی قرار گرفت. از میان مقالات یافت شده، مقالاتی که معیارهای ورود را داشتند، انتخاب و از سایر منابع مرتبط با آسیب‌شناسی گفتار و زبان نیز برای نگارش مقاله حاضر استفاده شد. یافته‌‌ها: از مجموع 50 مقاله به دست آمده در جستجوی اولیه، بر اساس معیارهای ورود ذکر شده 24 پژوهش جهت نگارش این مقاله مورد استفاده قرار گرفت. بعد از مطالعه عنوان و خلاصه مقاله‌ها و کتاب‌‌های مختلف از 24 مورد، مرتبط‌ترین آن‌ها جهت نگارش مقاله حاضر استفاده شد. نتیجه‌گیری: کودکان مبتلا به لکنت نسبت به کودکان طبیعی نقایصی در زمینه حافظه فعال نشان می‌دهند. مدل‌هایی که مربوط به وقوع و رشد لکنت می‌باشند، بهتر است بیشتر به نقش حافظه فعال بپردازند.
